# 577 Expanding Outpatient Burn Therapy Services to Address Social Determinants of Health

**DOI:** 10.1093/jbcr/irac012.205

**Published:** 2022-03-23

**Authors:** Susan L Smith, Rachel B Gonzalez, Cindy Mayorga, Howard G Smith

**Affiliations:** Orlando Health, Orlando, Florida; Orlando Health, Casselberry, Florida; Orlando Health- Warden Burn Center, Orlando, Florida; Orlando Health, Orlando, Florida

## Abstract

**Introduction:**

Occupational and Physical Therapist provide acute services to burn-injured patients and continue treatment well into their functional recovery for mobility, strengthening, scar management, contracture prevention and return of independence. Unfortunately, we recognized that patients encountered obstacles when attempting to access these services in the outpatient setting. It was evident that these impediments frequently coincided with social determinants of health (SDOH). According to Healthy People 2020, SDOH represent the cumulative influences of individual, cultural, community and societal attributes on health and quality-of-life. Self-pay patients accounted for 12% of clinic patients. However, patients with coverage were not always receiving adequate therapy due to lack of burn knowledge among community-based therapist. By expanding outpatient burn clinic services, we are better positioned to meet the needs of all burn-injured patients in our community.

**Methods:**

A plan was outlined to increase the presence of Occupational and Physical Therapy (OT/PT) in the outpatient burn clinic. Pertinent literature was reviewed, and a presentation was developed to establish need and outline a model for integration of services. The inpatient OT/PT department managers collaborated with the Burn Director and Burn APRN on projecting staffing and equipment needs. Multidisciplinary cost analysis was completed. Guidelines for outpatient burn rehabilitative therapy staffing and a description of services were developed.

**Results:**

After much collaboration and effort, and with community support, our PT/OT inpatient burn staff provide rehabilitative therapy to all patients attending outpatient burn clinic two days weekly. Additionally, educational material, hands on range of motion training and free new and donated compression garments are readily available during all four burn clinics weekly. The new outpatient burn clinic opened in 2021, equipped with two large showers and a designated therapy room, ready to wear compression garments, putty, and assistive devices designed to further support burn recovery. Attendance for burn outpatient visits increased significantly from 2015 to 2018.

**Conclusions:**

Transportation, childcare, employment restrictions, individual health and wellness beliefs, lack of funding and access to technology represent examples of blockades to healthcare care. This multidisciplinary effort increased the availability and scope of rehabilitative services in the outpatient burn clinic patient, allowing patients to receive comprehensive burn wound care and rehabilitative therapy in one location to promote healing, manage pain, improved function, maximizes independence and reintegrate into their family and community.

